# Effects of Fiber Inclusion on Pervious Concrete: A Multiscale Experimental and Imaging Study

**DOI:** 10.1021/acsomega.6c00238

**Published:** 2026-06-03

**Authors:** Demet Yavuz, Muhammed Serdar Avcı, Harun Alp, Şemsi Yazıcı

**Affiliations:** † Faculty of Engineering, Civil Engineering Department, 53000 Van Yüzüncü Yıl University, Bardakçı, Van 65080, Turkey; ‡ School of Civil Engineering, University College Dublin, Dublin DO4 V1W8, Ireland; § Faculty of Engineering, Civil Engineering Department, 37509 Ege University, Erzene Avenue, Izmir 30040, Turkey

## Abstract

As is known, pervious concrete is commonly used in low-traffic-load applications. Therefore, pervious concrete is exposed to flexural loads throughout its economic life. To improve the flexural behavior of pervious concretes, fibers are the most common solution used by researchers. Hence, fiber dosages of 0.1%, 0.2%, and 0.3% (by volume of concrete) are selected for this study. The influence of increased cement content is also discussed. Single-sized limestone aggregates without any mineral admixture were used to produce pervious concretes. The mechanical and hydraulic properties of the pervious specimens were examined. Additionally, image analyses using both CT (Computed Tomography) and scanned sliced samples were conducted. In this study, the samples containing 0.3% polypropylene fiber by volume yielded the highest splitting tensile and flexural strength. Adding fiber reduced the porosity of the pervious concrete. Mixtures with the highest fiber content exhibited the lowest porosity and permeability coefficients. Also, the porosity obtained from image analyses correlates well with volumetric porosity results. The impact of fiber additives on the strength and permeability of pervious concrete has been explored only to a limited extent in existing literature. This study contributes original insights by assessing this impact through a comprehensive approach that examines mechanical, hydraulic, and microstructural aspects, utilizing both experimental methods and image analysis.

## Graphical abstract

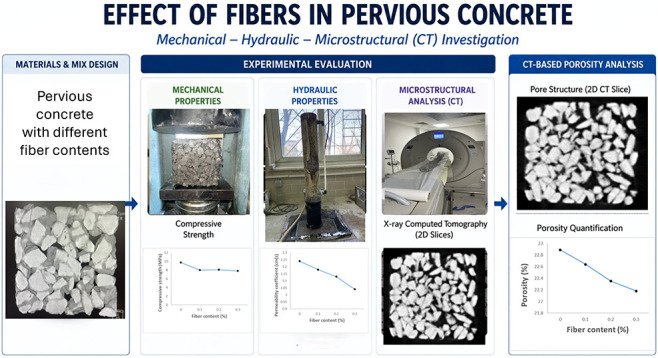

## 1 Introduction

Most of the urban areas are covered with impermeable pavements. With the growing population in these areas, the need for infrastructure such as roads, pavements, and parking lots is also increasing rapidly. As a result, a new pavement type has been adapted for urban use.^1–3^ Porous concrete can be produced with the same materials as conventional concrete. However, they differ in mechanical and hydraulic performances. These types of concrete contain large volumes of pores within their structure, mostly between 15% and 30%. These pores effectively drain stormwater from the surfaces of porous concrete into sublayers and groundwater.^4,5^ While exhibiting lower mechanical properties than traditional concrete, porous concrete has excellent water permeability performance as a direct result of its porous structure; these types of concrete also exhibit lower compressive and flexural strength. Porous concretes are commonly used in pavements, parking lots, and playgrounds, which are mostly subjected to flexural load. Because of these outcomes, fibers are incorporated into pervious concrete to improve flexural performance.^6–9^ Zhu et al.^10^ reported that, with an appropriate pervious concrete mix design, fibers can increase both the compressive and flexural strengths of pervious concrete. On the contrary, Tran et al.^11^ reported that fiber addition reduced both the compressive and flexural strengths of pervious concrete. Bright Singh and Madasamy^12^ used fibers at dosages up to 0.4%. They found that fiber dosage and type had no significant effect on the porosity and permeability of pervious concrete.

Since achieving a highly porous structure is a key factor in ensuring effective drainage in pervious concrete, determining porosity and its relationship with water permeability has recently attracted considerable attention from researchers worldwide. While determining total porosity is essential, it has been reported that the water permeability of porous concrete is usually related to connected porosity.^13^ Also, the distribution of pores, their shapes, and sizes play an important role in water permeability.^14,15^ Image analysis has been used for the past two decades to characterize the morphology and distribution of pores in porous concrete.^16^ Image analysis can be carried out using Computed Tomography (CT) and X-ray tomography, as well as scanned images.^17–20^ CT or X-ray tomography can readily detect pore distribution in all porous structures. Image analysis with scanned images involves sectioning the porous sample chiefly into slices. Using these slices, porosity, pore sizes, and their distribution can be investigated. Image analysis can also be used to define clogged sections, as reported by researchers such as Kayhanian et al.^21^ and Deo et al.^22^

Researchers have proposed various solutions to improve the performance of concrete mixes. It has been reported that replacing cement with rice husk ash at a rate of 5% increased the 28-day compressive strength by 2.4%.^23^ Şengel et al.^24^ reported that using waste steel rubber wire in concrete reduced maximum deformation while yielding promising results in terms of impact resistance. Similarly, studies indicate that using different fibers in concrete improves mechanical properties.^25–30^

Determining the proper dosage of fibers in pervious concretes should be done carefully because these types of concretes contain lower cement paste compared to traditional concretes. Therefore, covering fibers in a cement matrix can be challenging. The addition of fiber to pervious concrete remains underexplored. Liu et al.^31^ and Juradin et al.^32^ reported that fibers generally do improve the mechanical properties of concretes, while Kevern et al.^33^ reported that the involvement of macro synthetic fibers leads to decreased permeability. Alemu et al.^34^ used different aggregate types and poly­(vinyl alcohol) (PVA) fibers with lengths of 12–13 mm. It was found that while synthetic fibers prevent the sagging of cement paste, exceeding 0.5% (wt cement) results in higher total porosity and lower compressive strength. On the other hand, Rangelov et al.^35^ reported that solidified carbon fibers had a positive effect on both the permeability and mechanical properties of pervious specimens. Zhai et al.^36^ used basalt and PVA fibers in pervious concrete slabs and reported that adding 0.2% basalt fiber to the pervious mix initially decreased compressive strength. The compressive strength of the specimens was the highest at a 0.3% fiber dosage. Wang et al.^37^ used basalt fibers of four lengths (12, 15, 18, and 24 mm) and reported that the compressive strength of pervious concrete increased with increasing basalt fiber length. Wu et al.^38^ noted that basalt fiber addition initially increased the compressive strength of pervious concrete, after which it decreased. They also reported that the presence of fibers improved acid corrosion resistance compared with pervious specimens without fibers. Ozel et al.^39^ added steel and polypropylene fibers to the pervious concrete. They reported that a pervious mixture with steel fibers exhibited higher abrasion resistance, whereas mixes with polypropylene fibers improved permeability.

Researchers have used image analysis methods to characterize concretes over the years. It can be used to characterize, crack propagation,^40^ determine the porosity of concrete,^41^ assess compressive strength of traditional concrete,^42^ monitor shrinkage strains,^43^ perform durability testing,^44^ and detect pinholes in concrete surfaces.^45^ Hutchinson and Chen^46^ used image analysis to assess structural damage in concrete; Fonseca and Scherer^47^ used it to characterize air voids in concrete and mortar. In pervious concrete, porosity and void structures can also be quantified using image analysis.^48^ Image analysis in pervious concretes can be performed by using Computed Tomography (CT) or scanned images.^48,49^ In this study, both methods were used and compared. To determine the porosity of pervious concrete using scanned images, samples are sliced to specific thicknesses, limiting the number of samples that can be obtained from a single piece. The resulting sections are then scanned with a camera or scanner and processed using software (e.g., Image-Pro) to render voids in black and the filled areas (cement paste and aggregates) in white.^49^ Some researchers have proposed using different materials to facilitate the separation of pores from the filled areas.^50^ In contrast, computed tomography (CT) is a preferred method because it can nondestructively determine the internal structure of samples. Researchers often use CT to obtain both 2D and 3D pore structures. This technique captures numerous images of pervious concrete at predetermined intervals, which can then be used in various software (e.g., Avizo) to generate 3D models of the samples.^51^

A review of the studies summarized above indicates that researchers have extensively investigated the mechanical and hydraulic properties of pervious concrete. However, the effects of varying fiber concentrations on the internal structure of pervious concrete, using techniques such as computed tomography (CT) and cross-sectional image analysis, have not been adequately explored. Therefore, this study aims to experimentally examine the mechanical and hydraulic properties of pervious concrete reinforced with fibers and to determine and compare the total porosity of the samples using image analysis. Thus, pervious mixes with three different fiber dosages, namely 0.1%, 0.2%, and 0.3% (by volume), are used to investigate the properties of pervious concrete. Compressive, splitting tensile, and flexural strength, porosity, and water permeability coefficients are studied. The effect of fiber volume on such properties is widely discussed. Porosity was determined by image analysis in addition to the experimentally obtained results. Therefore, both computed tomography (CT) and scanned sections were used. Approximately 200 CT slices were obtained from pervious concrete, and the total porosity was calculated from these slices. In addition, a porosity value was determined from 10 images obtained by scanning the cylinder samples into five equal parts and comparing them.

## 2 Experimental Program

### 2.1 Materials

CEM I 42.5 R type ordinary cement following TS EN 197-1 ^52^ was used. The specific gravity of the cement is 3.15. The pervious concrete mix was designed without fine aggregate. Limestone aggregates with a 15–25 mm fraction were used as coarse aggregates. To minimize moisture content variation, all aggregates were used under surface-dry (SSD) conditions. The properties of aggregates are listed in Table 1. A superplasticizer (SP) admixture was used in several mixtures with varying fiber dosages. Macro polypropylene fibers (PP) with a length of 54 mm were used. Properties of PP fibers are presented in Table 2, and visuals of these fibers are presented in Figure 1. The gradation curve of limestone aggregate is presented in Figure 2. Pervious concrete differs from traditional concrete by intentionally using single-sized or gap-graded aggregates to create an interconnected void structure. As a result, adhering to the upper and lower grading limits set by standards is not a primary design requirement. Instead, the grading of the aggregates is chosen specifically to achieve the desired levels of porosity and permeability. Therefore, in this study, the gradation curve does not present such grading limits.

**Table 1:** Physical Properties of Limestone Aggregates

Physical properties	15–25 mm aggregates
Specific gravity	2.71 ± 0.02
Water absorption (%)	0.23 ± 0.01

**Table 2:** Properties of Fibers

Macro fiber	Characteristic features
Density (g/cm^3^)	0.91
Length (mm)	54
Diameter (mm)	0.95
Tensile stress (MPa)	530
Modulus of elasticity (GPa)	7.2
Melting point (°C)	160

**Figure 1:**
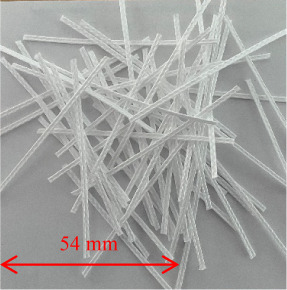
Polypropylene fibers.

**Figure 2:**
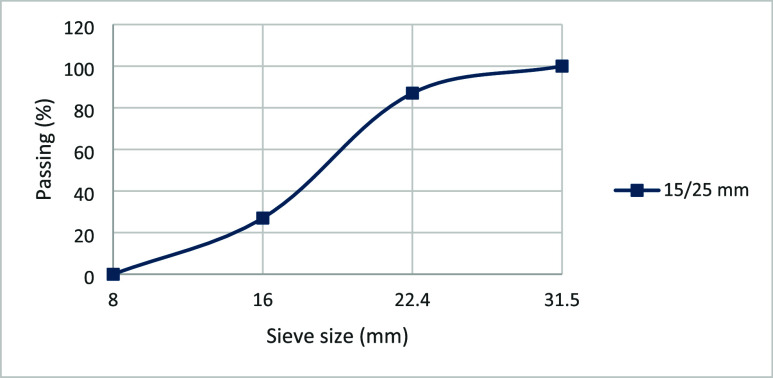
Gradation curve of 15/25 mm limestone aggregates.

### 2.2 Concrete Mix Design

Single-sized limestone aggregates were used in the pervious mix design. In pervious concretes produced using fibers, determining the amount of cementitious material is crucial. Excessive cementitious paste will cause it to flow and clog voids, while insufficient paste will prevent the concrete from achieving the desired performance because it cannot coat the aggregates and fibers. While the literature indicates a minimum cementitious material usage of 150 kg/m^3^ and a maximum of 560 kg/m^3^, in this study, based on the literature and researchers’ own experience, the usage was set at 300 kg/m^3^ and 350 kg/m^3^, respectively.^20,35,53,54^

This was done to ensure that sufficient cement paste was present in the system to wrap the fibers at the specified fiber dosages, and to prevent excess cement paste from flowing downward and clogging the concrete voids. Water/cement ratios were kept constant at 0.35. The target porosity of the pervious mixes was 20%. Polypropylene fibers with a length of 54 mm were added to the mixture at three dosages (by volume): 0.1%, 0.2%, and 0.3%. At the highest fiber dosage (0.3%), superplasticizer (sp) was added to obtain the desired workability. All the mixtures showed zero slump, a characteristic of pervious concrete due to its low paste content and lack of fine aggregates. The structure of open-graded aggregates ensures internal stability, preventing significant deformation under gravity. Adding 54 mm fibers (at 0.1–0.3%) enhanced internal friction and interlocking, resulting in a higher static yield stress for the mixture. The slump values for all mixes were recorded as zero. Theoretical mix designs for pervious concrete are listed in Table 3, and revised mix ratios are presented in Table 4.

**Table 3:** Theoretical Mix Ratios for Pervious Mixes (1 m^3^)

Mix	Cement (kg)	Water (kg)	Aggregates (kg)	Superplasticizer (kg)	Fiber (%)
300–15/25 mm-Control	300	105	1625	-	-
300–15/25 mm-0.1%	300	105	1625	-	0.1
300–15/25 mm-0.2%	300	105	1625	0.1	0.2
300–15/25 mm-0.3%	300	105	1625	0.2	0.3
350–15/25 mm-Control	350	122.5	1535	-	-
350–15/25 mm-0.1%	350	122.5	1535	-	0.1
350–15/25 mm-0.2%	350	122.5	1535	0.1	0.2
350–15/25 mm-0.3%	350	122.5	1535	0.2	0.3

**Table 4:** Revised Mix Ratios for Porous Concrete (1m^3^)

Mix	Cement (kg)	Water (kg)	Aggregates (kg)	Superplasticizer (kg)	Fiber (%)
300–15/25 mm-Control	290	101.5	1571	-	-
300–15/25 mm-0.1%	292	102.1	1580	-	0.1
300–15/25 mm-0.2%	292	102.0	1579	0.1	0.2
300–15/25 mm-0.3%	293	102.4	1585	0.2	0.3
350–15/25 mm-Control	339	117.8	1487	-	-
350–15/25 mm-0.1%	341	119.5	1497	-	0.1
350–15/25 mm-0.2%	342	119.5	1498	0.1	0.2
350–15/25 mm-0.3%	342	119.6	1498	0.2	0.3

### 2.3 Methods

Pervious concrete compaction was performed using a standard rod. First, fresh concrete was placed in the mold to fill 1/3 of the mold, and the mold was rodded 25 times. Next, two-thirds of the mold was filled and rodded 25 times. The remaining portion of the mold was then filled with concrete, rodded 25 times, and left in the mold for 1 day. After demolding, the samples were cured for 28 days. Specimen dimensions and the standards followed are listed in Table 5. The density of freshly poured pervious concrete was determined in accordance with ASTM C1688.^55^ For porosity measurement, specimens were submerged, and the volume of displaced water was calculated. With this method, the volumetric porosity of pervious specimens was determined as the percentage of the void volume. Two porosities were determined: total and effective. Samples were removed from the curing chamber and placed at room temperature for 1 min to ensure discharge of the connected pores. The volume and the weight of the specimen were noted. Then, pervious specimen was immersed in a water-filled container for 24 h. After that, specimens were dried at 110 °C and weighed. The following equations were used to calculate the effective and total porosity of pervious concretes, respectively.

1 $$P_{e}=\left(1-\frac{w_{3}-w_{1}}{V\times \rho_{w}}\right)\times 100\%$$

2 $$P_{t}=\left(1-\frac{w_{2}-w_{1}}{V\times \rho_{w}}\right)\times 100\%$$

where *P*_e_ is the effective and *P*_t_ total porosity, *w*_1_ is the weight of the specimen immersed in water (24 h in g), *w*_2_ is the weight of the specimen dried at 110 °C (g), *w*_3_ is weight of cured specimens (g), *V* is the specimen volume (cm^3^) and *ρ*_w_ density of water (g/cm^3^).

**Table 5:** Standards and Specimen Sizes

	Specimen size (mm)	Standard
Fresh density	-	ASTM C1688 ^55^
Volumetric porosity	100 × 200	Alemu et al.^56^
Compressive strength	150 × 150 × 150	TS EN 12390-3 ^57^
Splitting tensile strength	100 × 200	TS EN 12390-6 ^58^
Flexural strength	100 × 100 × 400	TS EN 12390-5 ^59^
Permeability coefficient	100 × 200	Tran et al.^11^
Porosity with image analysis (CT)	100 × 100 × 100	Yu et al.^19^
Porosity with image analysis (sectioned pieces)	100 × 40	Neithalath et al.^48^

A falling-head permeameter was used to determine the permeability coefficient (*k*). In this permeameter, the first specimen was confined with a waterproof material and placed in the test setup shown in Figure 3. Water was introduced into the system, and the time required for it to travel 220 mm was recorded with a stopwatch. And eq 3 was used to calculate *k*;

3 $$k=\frac{Q\times L}{H\times A\times t}$$

where *Q* is the discharged water (mm^3^), *L* is specimen height (mm), *A* is the surface area of the pervious specimen (mm^2^), *t* is time (s), and *H* is water height (mm).

**Figure 3:**
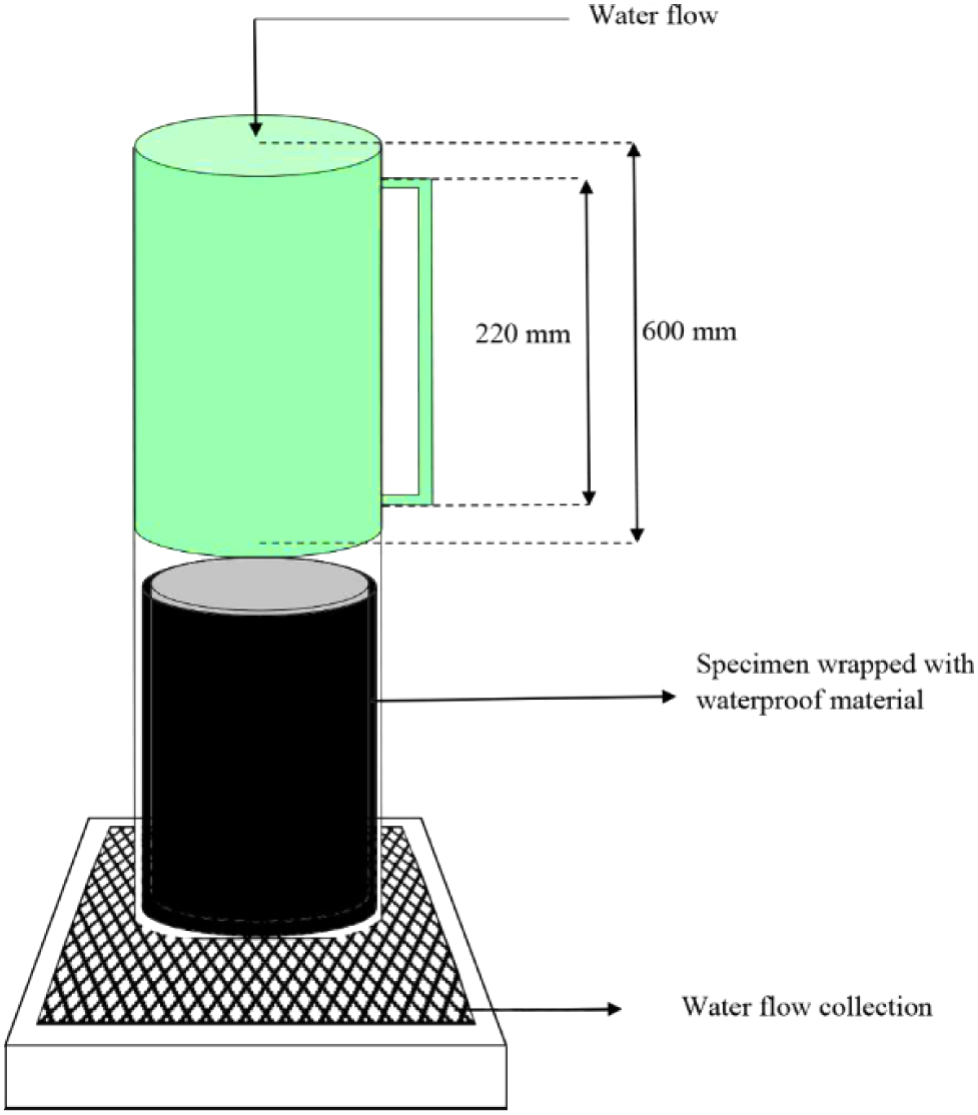
Test setup for permeability coefficient.

Compressive, splitting tensile, and flexural strength were determined in accordance with TS EN 12390-3, TS EN 12390-6, and TS EN 12390-5, respectively. Related formulas are given below.

4 $$\sigma_{s}=\frac{2P}{\pi LD}$$

where σ_s_ is the splitting tensile strength (MPa), *P* is the maximum load at fracture (N), *L* is the length of the specimen (mm), and *D* is the diameter of the cylinder (mm).

5 $$\sigma_{f}=\frac{3PL}{2bd^{2}}$$

where σ_f_ is the flexural tensile strength (MPa), *P* is the maximum load at fracture (N), *d* is the distance between supports (mm), *b* is the width of the beam (mm), and *h* is the height of the beam (mm).

Porosity was also calculated using image analysis. For image analysis, two different processes were used. The first method for obtaining image acquisition was computed tomography (CT). Over 200 different slices were attained, and the porosity of the pervious concretes was calculated. The steps followed are shown in Figure 4. Moreover, the cylinder-pervious concrete was sectioned into 40 mm slices, yielding a total of 5 pieces. Furthermore, the cut specimens were scanned front and back (a total of 10 images), and the porosity in the images was quantified. Then, both results were compared to volumetric porosity results. A detailed explanation of the following steps is illustrated in Figure 5.

**Figure 4:**
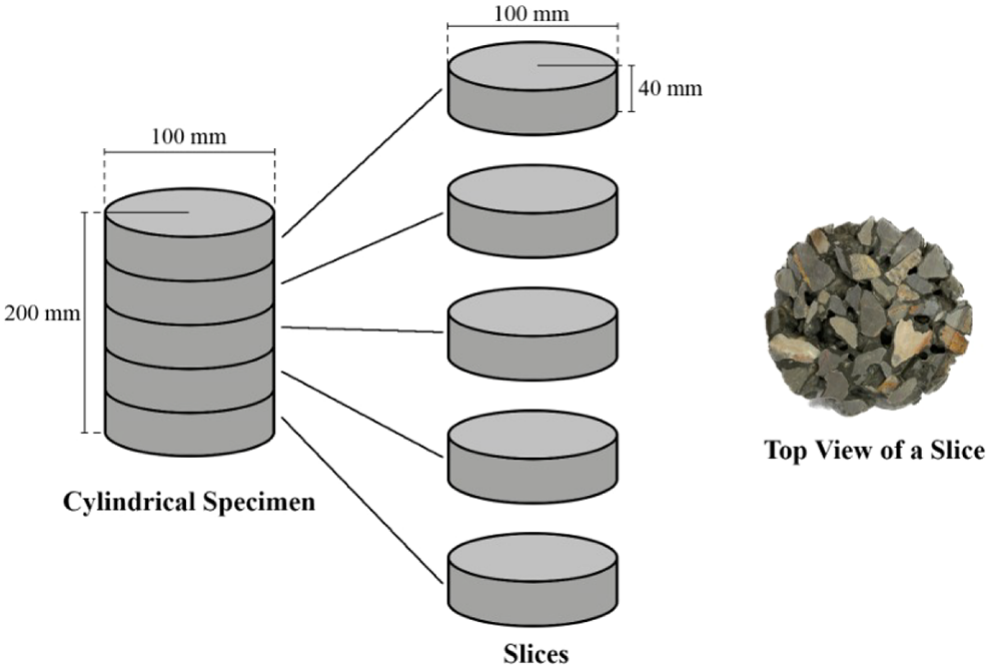
Cutting process of cylinder specimens for image analyses.

**Figure 5:**
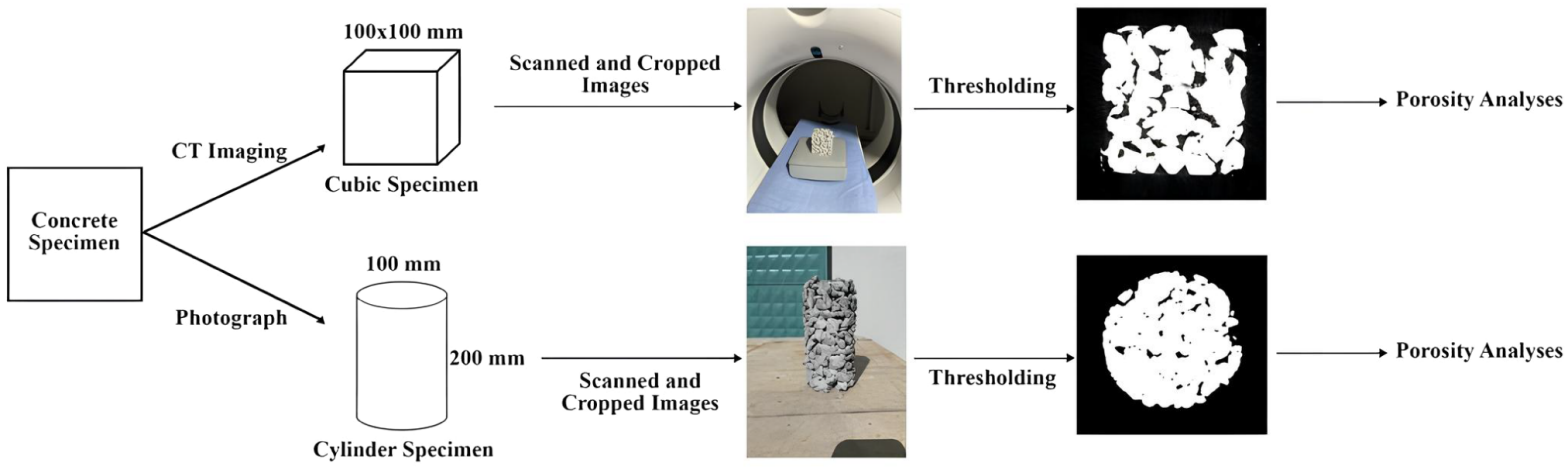
Image analysis procedure for porosity quantification.

Porosity was calculated directly from digital images through a sequence of preprocessing, segmentation, and quantification steps. Each RGB image, denoted I_“RGB” (*x*,*y*)­(*x*,*y*), was first converted to grayscale to reduce complexity. The grayscale intensity was obtained as a weighted sum of the red, green, and blue channels:

6 I(x,y)=0.299R(x,y)+0.587G(x,y)+0.114B(x,y)

To suppress image noise, the grayscale image was smoothed using a Gaussian filter,

7 $$Ĩ\left(x,y\right)=\left(I\times G_{\sigma }\right)\left(x,y\right)$$

where *G*_σ_ is a Gaussian kernel of standard deviation σ.

Segmentation was then performed to distinguish pores from the solid matrix. A global threshold *T* was applied to generate a binary image,

8 $$M\left(x,y\right)=\{\begin{array}{ll} 1, & Ĩ\left(x,y\right)\leq T,\\ 0, & Ĩ\left(x,y\right)>T \end{array}$$

where pixels with value 1 correspond to pore space. The total pore area in pixels was determined as,

9 $$A_{\mathrm{pore}}^{\mathrm{px}}=\underset{x,y}{\sum }M\left(x,y\right)$$

while the total analyzed area was simply the image size,

10 $$A_{\mathrm{tot}}^{\mathrm{px}}=W\times H$$

with *W* and *H*, the image width and height in pixels. Finally, global porosity was calculated as the ratio of pore area to total area,

11 $$\phi =\frac{A_{\mathrm{pore}}^{\mathrm{px}}}{A_{\mathrm{tot}}^{\mathrm{px}}}$$

and reported as a percentage,

12 Porosity(%)=100×ϕ

A detailed workflow for the following segmentation steps is presented in Figure 6.

**Figure 6:**
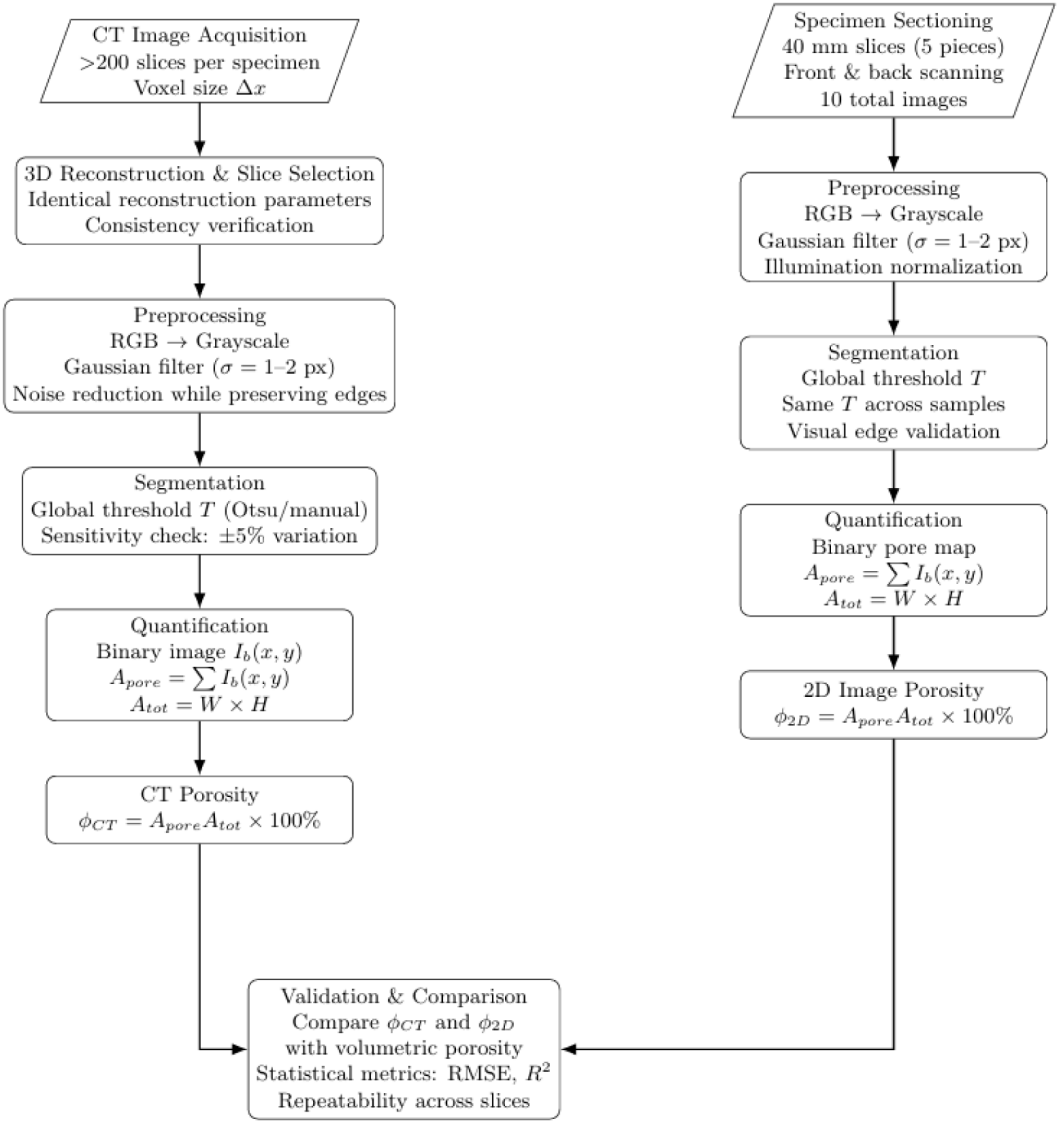
Workflow of image processing.

CT scanning was performed using a medical CT scanner. Scans were conducted at a tube voltage of 120 kV and tube current of 200–250 mA under automatic exposure control. The rotation time was set to 1.0 s. Images were obtained with a slice thickness of 1.0 mm.

## 3 Results and Discussion

### 3.1 Density

Pervious concretes have a lower density than conventional concrete due to the intentionally created voids within them to allow water to pass through. Ibrahim et al.^60^ stated that the density of pervious concretes is proportional to porosity; in their study, they reported that pervious concrete with 37% porosity has a density of approximately 1716 kg/m^3^. Researchers have reported that decreasing density increases concrete porosity, thereby reducing mechanical properties.^61^ Therefore, determining the density of pervious concretes is quite important. The density values of pervious concretes are presented in Figure 7. As shown in Figure 7, the density values of the pervious mixes ranged from 1983 to 2027 kg/m^3^. Fiber addition had little effect on the density of pervious concrete. Polypropylene fiber addition had no significant effect on density because of its low volume fraction in the matrix.

**Figure 7:**
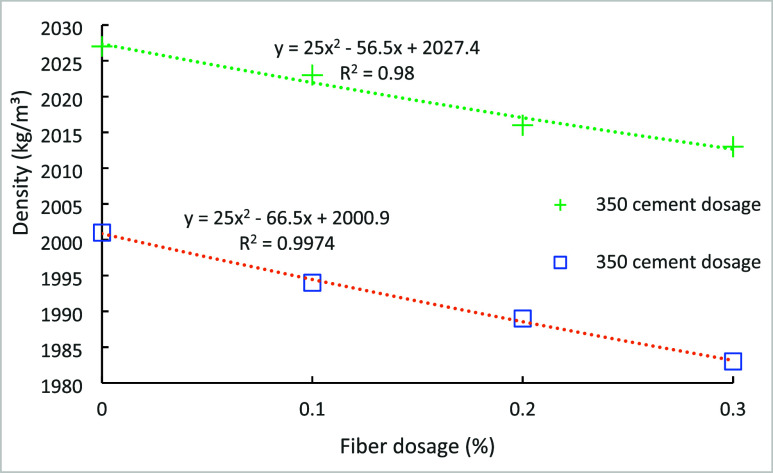
Density of pervious concrete.

### 3.2 Compressive, Splitting Tensile, and Flexural Strength

Compressive strength test results are presented in Table 6. The compressive strength of pervious concretes varied between 7.82 and 16.36 MPa. Since pervious concrete with lower compressive strength can be used in low-traffic load environments, the pervious mixes designed in this study can be used in walkway construction. The compressive strength test setup is presented in Figure 8.

**Table 6:** Mechanical Strength Test Results

Mix	Compressive strength (MPa)	Standard deviation	Splitting tensile strength (MPa)	Standard deviation	Flexural strength (MPa)	Standard deviation
300–15/25 mm-Control	9.73	0.72	0.63	1.11	2.16	0.95
300–15/25 mm-0.1%	8.00	0.81	0.65	1.26	2.25	1.07
300–15/25 mm-0.2%	8.10	0.86	0.68	1.14	2.47	0.92
300–15/25 mm-0.3%	7.82	0.88	0.70	1.32	2.64	1.12
350–15/25 mm-Control	16.36	0.84	1.80	1.06	3.82	1.02
350–15/25 mm-0.1%	16.01	0.91	1.88	1.17	4.19	1.15
350–15/25 mm-0.2%	16.11	0.96	1.97	1.23	4.37	1.09
350–15/25 mm-0.3%	15.44	1.03	2.17	1.28	4.71	1.22

**Figure 8:**
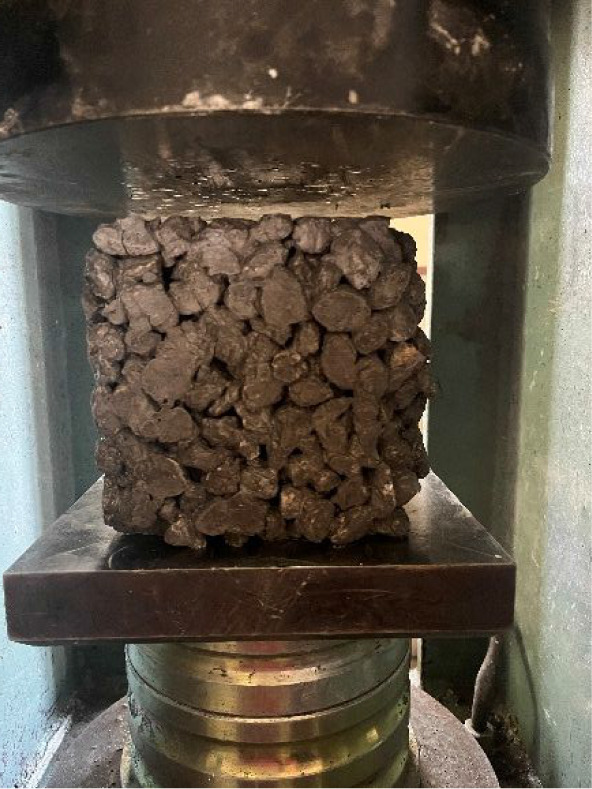
Compressive strength test setup.

As expected, increased cement content resulted in higher compressive strength. Some researchers have reported that fiber addition reduces the compressive strength of pervious concrete.^45,62^ Increasing the cement content increases the amount of paste surrounding the coarse aggregates, thereby improving the bond between the coarse aggregates and the paste. Additionally, a higher volume of cement paste leads to a less porous internal structure. This increased paste also fills the voids between the aggregates, resulting in more compact concrete.^63^ The findings of this study also support these arguments. In this study, although compressive strengths decreased with a 300 kg cement dosage, strengths were comparable in those produced with a 350 kg dosage. This behavior may be caused by insufficient cement paste to wrap the fiber when the fiber is used in pervious concrete produced with low cement dosage. When fiber is used in pervious concrete with a low cement dosage, the fiber cannot exhibit its beneficial effect because there is insufficient cement paste to encapsulate it. However, as the amount of paste increases, the fibers can also exhibit a filling effect, as they are wrapped in cement paste. It is thought that this is the cause of the mentioned behavior.

Cement paste, as is well-known, acts as a glue, binding the aggregates and fibers within the concrete. However, when low cement dosages are used, the cement paste in the system becomes less dense and weaker (less C–S–H gel formation). It loses its ability to fully encapsulate aggregates and fibers. This results in a more porous and cohesive matrix, thus limiting the strength of concretes. Furthermore, in mixtures produced with low cement amounts, sufficient hydration products may not form at the interface transition zone, the weakest region of the cement paste, to create a strong interface. Reducing the cement paste volume to fill voids also increases paste porosity, thereby decreasing compressive strength.^64–66^

Splitting tensile strength of pervious varied from 0.63 to 2.17 MPa, as shown in Table 6. Increasing the cement content and adding fibers improved splitting tensile strength across all mixes. Maximum improvement was achieved with a 0.3% fiber volume at a cement dosage of 350 kg, followed by the exact fiber dosage and 300 kg cement, with 11.11%. Because of their bridging effect, polypropylene fibers are known to increase the splitting tensile strength of pervious concretes.^39^ As shown in Figure 9, there is a linear relationship between compressive and splitting tensile strength.

**Figure 9:**
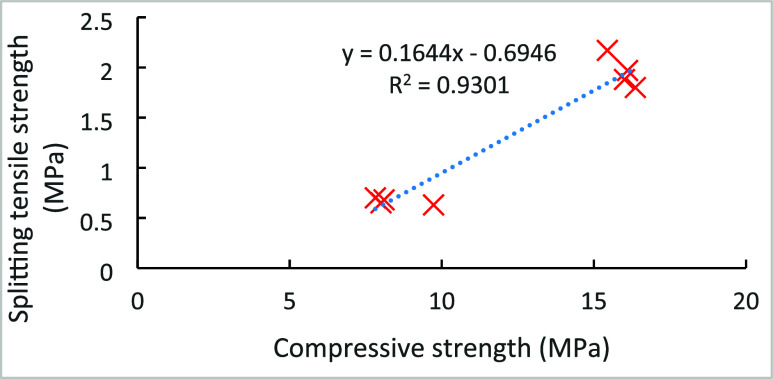
Relationship between compressive and splitting tensile strength.

The flexural strength of pervious concretes ranged between 2.16 and 4.71 MPa. Previous studies have reported that fiber addition decreases the flexural strength of pervious concrete.^11^ Contrary to that, the findings of this study show that fibers lead to an increase in the flexural strength of pervious concretes. Similar to the tensile strength, the maximum increase was observed at 23.30% at a fiber dosage of 0.3%. With increasing cement dosage, there is enough cement paste in the permeable concrete to wrap both the aggregate and the fibers. Thus, with the use of fibers, a bridging effect is observed, leading to increased bending strength. The relationship between splitting tensile and flexural strength is given in Figure 10. The appearance of the concrete sample after splitting tensile strength is shown in Figure 11.

**Figure 10:**
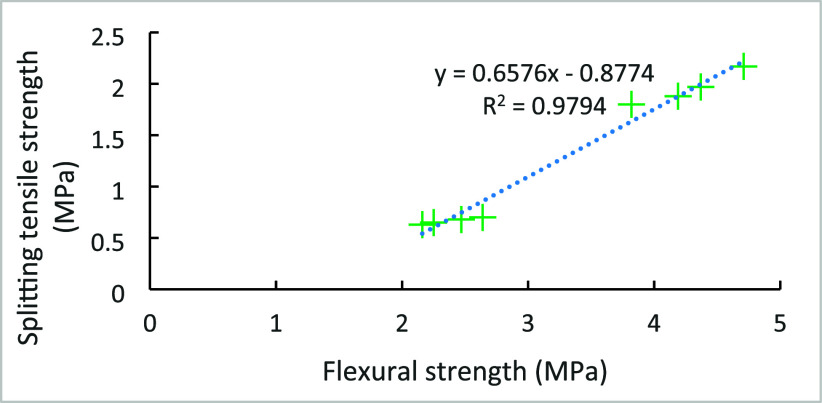
Relationship between splitting tensile and flexural strength.

**Figure 11:**
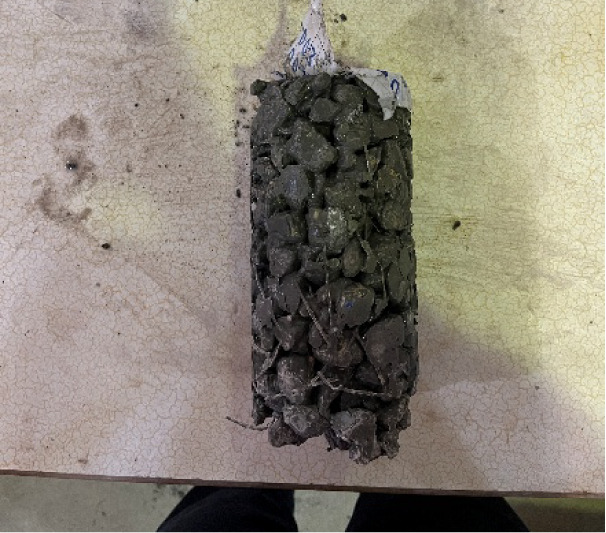
Pervious concrete sample after the splitting tensile test.

In conventional concretes, the use of fibers generally results in an improvement in concrete strength. Due to the homogeneous distribution of fibers in the concrete mixture, a bridging effect occurs, thereby improving concrete strength. However, previous studies on pervious concrete have reported different results. It is reported that this behavior is caused by the fact that pervious concretes contain much larger voids than conventional concretes.^67^ Improvements of up to 47% have been reported for some fiber types in the literature.^68^ However, there are studies indicating that with increasing amounts of fiber use, the compressive strength decreases,^11,69^ increases,^56,70^ or increases initially but starts to decrease as the fiber volume continues to increase.^33,71^ The flexural strength of pervious concretes is generally reflected positively in the results. It was stated that adding fibers can improve binder performance. Hence, the mechanical properties of permeable concretes. However, to determine their behavior under different curing conditions, Li et al.^72^ reported that low dosages of polyethylene fibers showed better performance than other curing methods, achieving 22.47 MPa flexural strength at a 0.7% dosage under 48 h steam curing and 48 h hot-water bath curing. In another study,^39^ increases in flexural strength of up to 115.7% were achieved by using metal fibers.

Even at low volumetric dosages, the addition of long PP fibers (54 mm in length) to the concrete mix creates additional surface area that must be covered by the cement paste. This has led to reductions in compressive strength, especially in mixes produced with lower cement quantities. With higher cement quantities, a bridging effect was observed due to the increased fiber volumetric use. Specifically, in flexural and tensile strength, it was observed that using 0.3% fibers resulted in fibers extending throughout the concrete sample and bonding extensively with one another, thereby improving these characteristics.

### 3.3 Volumetric Porosity

Volumetric porosity results (both total and effective) are given in Figure 12. As expected, effective porosity was lower than total porosity across all mixes. While total porosity affects the compressive strength of pervious concrete, its drainage capacity is directly related to effective porosity.^11^ Therefore, in this study, we focused on the most effective porosity of pervious concrete. When the effective porosity of pervious concretes was analyzed, it was observed that the addition of fibers slightly decreased the porosity values. Increasing cement content also negatively affected the porosity of pervious concretes. The effective porosity of pervious concrete decreased by 1.09%, 1.70%, and 2.83% compared with the control specimen with 300 kg of cement content with PP fiber additions of 0.1%, 0.2%, and 0.3%, respectively. Moreover, for a 350 kg decrease in cement content, the effective porosity values were 1.57%, 3.29%, and 5.16% for PP fiber additions of 0.1%, 0.2%, and 0.3%, respectively. Ozel et al.^39^ reported that fiber addition increased the porosity of pervious concretes due to weak areas in the pervious matrix. Similar results were reported by Nassiri et al.^9^ The researchers reported that the porosity of the samples containing carbon fiber was higher than that of the control mixtures. Contrary to these results, Bright Singh and Madasamy^12^ reported that the porosity of pervious concrete samples decreased with the incorporation of carbon fiber. Wu et al.^38^ reported that the porosity of pervious concretes decreased with the use of increased amounts of basalt fiber. The findings of this study agree with those of Tran et al.,^11^ who reported that fiber addition decreased the porosity of pervious concretes.

**Figure 12:**
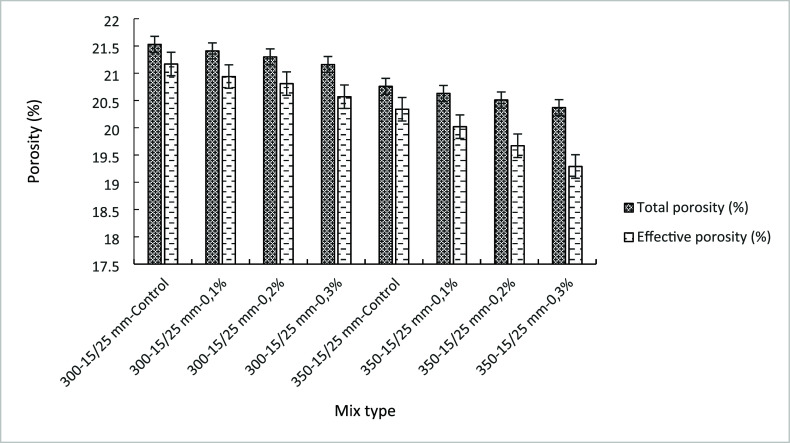
Total and effective porosity results.

### 3.4 Permeability Coefficient

It is not possible to classify concretes that do not provide the desired water permeability as pervious concrete. For this reason, the water permeability coefficients of pervious concrete should be determined, thereby enabling assessment of their capacity to drain water quickly. Researchers have reported that the water permeability coefficient is directly related to the porosity of pervious concretes.^73,74^ Therefore, permeable concrete with high porosity is expected to have a higher water permeability coefficient. The key point is that the interconnected voids facilitate water transmission between voids in pervious concrete. Thus, the connected porosity value rather than the total porosity affects the water permeability coefficient.^75^ Permeability coefficients of pervious concretes investigated are illustrated in Figure 13. As shown in Figure 13, increased cement content decreased the permeability coefficient, consistent with the decrease in effective porosity. The highest permeability coefficient was observed in control specimens with lower cement content (300 kg), at 1.24 cm/s. As fiber dosage increased, the permeability coefficient decreased. Fibers fill the voids within the pervious structure, thereby reducing its permeability. Figure 14 illustrates the relationship between effective porosity and the permeability coefficient. The permeability coefficients of pervious concrete samples increased with increasing effective porosity. In this study, a strong correlation (*R* ^2^ = 0.88) was observed between effective porosity and the permeability coefficient. The relationship between effective porosity and permeability was modeled using a power function rather than a linear regression. This selection is based on classical porous media theory (Kozeny–Carman), which describes permeability as a nonlinear function of porosity. In pervious concrete, increases in effective porosity enhance pore connectivity and reduce tortuosity, thereby increasing flow capacity. Therefore, a power-law model more accurately represents the physical transport mechanism than a linear approximation.

**Figure 13:**
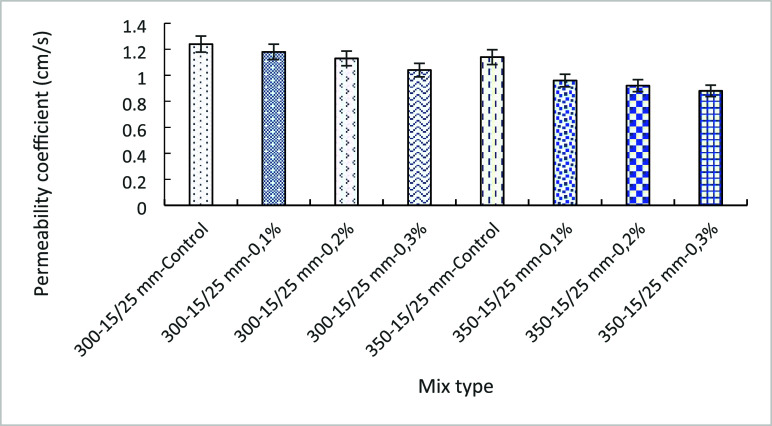
Permeability coefficients of pervious concrete.

**Figure 14:**
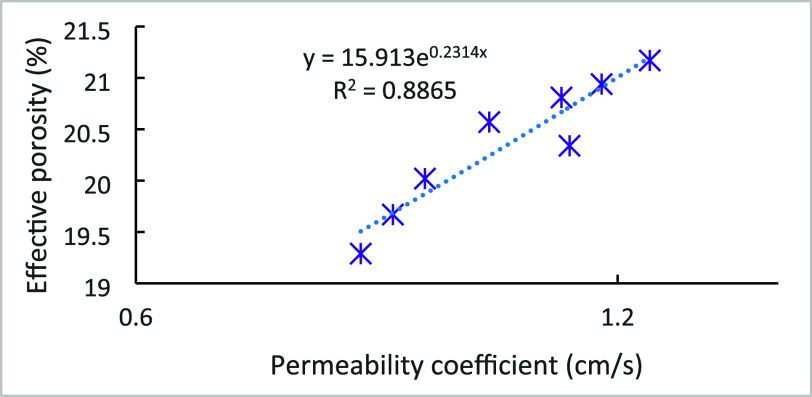
Relationship between effective porosity-permeability coefficient.

### 3.5 Image Analyses

Using image analysis, the porosity of pervious concrete can be determined quickly and easily without damaging the concrete. In addition, the void distribution in permeable concrete can be obtained cross-sectionally.^76,77^ Total porosity results obtained from the volumetric method, CT, and scanned images are listed in Table 7. Both raw and binary CT images of pervious concretes for all samples are presented in Figure 15. The porosity of pervious concrete directly affects its performance. In this study, porosities of pervious concrete were measured using three methods and compared. Thus, the aim is to determine whether differences exist among porosity measurement methods and to guide future studies. As shown in Table 7, the porosity results obtained from image analyses (CT and scanned images) are in acceptable agreement with the corresponding volumetric porosities. Other studies have reported that, when a sufficiently large number of random samples are tested, the area fractions of these samples equal volumetric porosity.^17^ Porosity results obtained with image analyses are slightly higher than those obtained from volumetric porosity measurements. Errors can occur during the thresholding of raw images.^17,48^

**Table 7:** Porosity Results of Pervious Concretes with Different Methods

Mix	Volumetric porosity (%)	Standard deviation	Area fractions of CT images (%)	Standard deviation	Area fractions of scanned images (%)	Standard deviation
300–15/25 mm-Control	21.53	0.75	22.89	0.84	22.61	0.66
300–15/25 mm-0.1%	21.41	0.77	22.64	0.71	22.43	0.83
300–15/25 mm-0.2%	21.30	0.88	22.35	0.92	22.17	0.76
300–15/25 mm-0.3%	21.16	0.92	22.18	0.77	22.03	1.09
350–15/25 mm-Control	20.76	0.82	21.63	0.85	21.86	0.84
350–15/25 mm-0.1%	20.63	0.66	21.26	0.94	21.43	0.76
350–15/25 mm-0.2%	20.51	0.92	21.06	0.79	19.92	0.93
350–15/25 mm-0.3%	20.37	1.11	20.73	0.88	19.50	0.87

**Figure 15:**
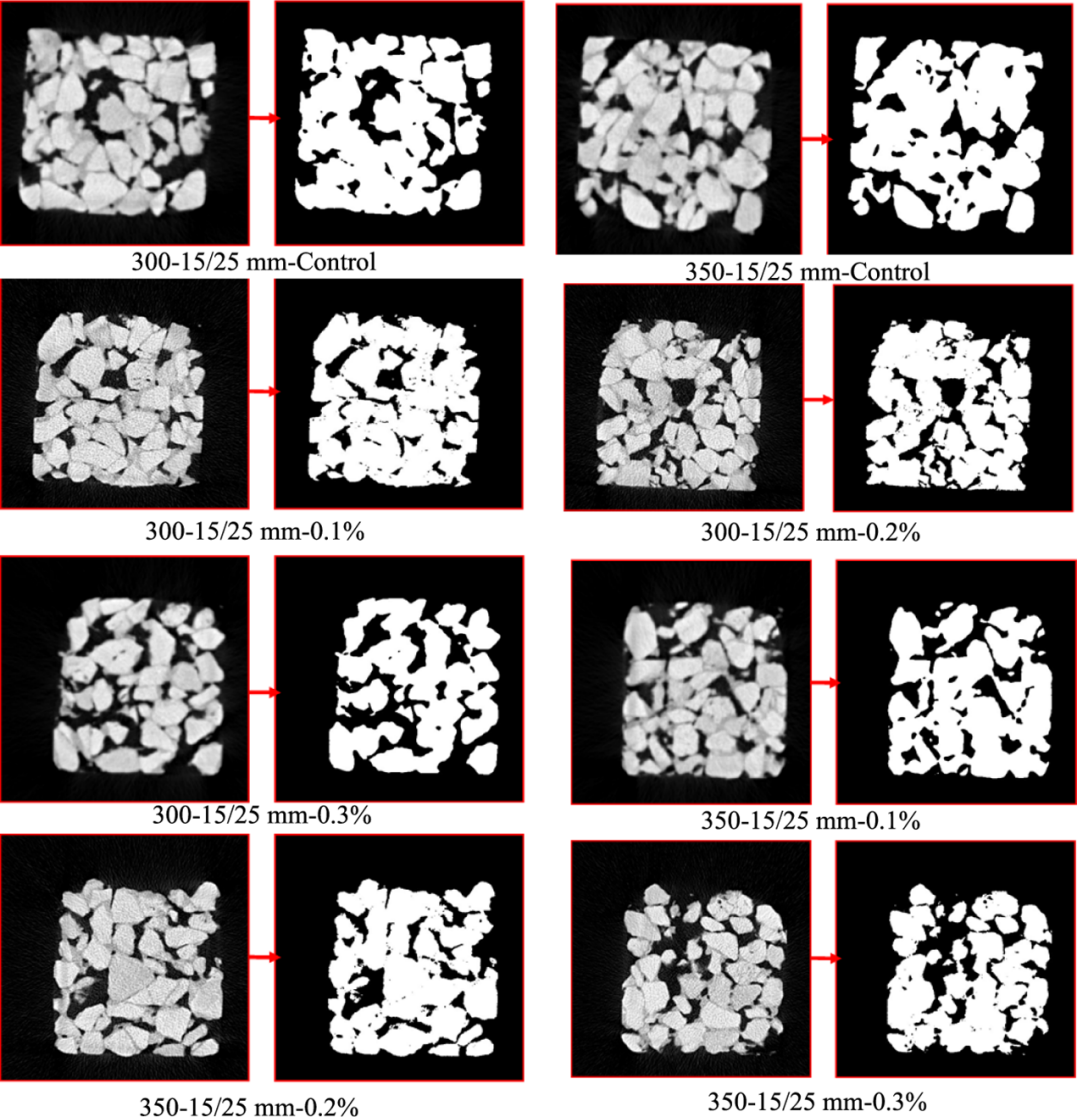
Raw and binary CT images of pervious mixes.

## 4 Conclusions

A detailed investigation was conducted into pervious concrete containing fibers at different volumetric fractions. The following conclusions are made:

- The fresh density of pervious concretes is slightly affected by fiber volume.
- Regarding mechanical properties, PP fiber addition caused decreased compressive strength. Contrary to this result, the flexural and splitting tensile strength of pervious concretes increased with increased fiber dosage. The highest results were obtained at a fiber dosage of 0.3% for flexural and splitting tensile strengths, with values of 4.71 and 2.17 MPa, respectively.
- The pervious concretes incorporating PP fibers showed lower porosity and permeability results compared to control samples. Moreover, the lowest permeability and effective porosity were observed at a fiber volume of 0.3%, with values of 0.88 cm/s and 19.29%, respectively.
- The porosity of pervious concretes calculated with CT and scanned images with sectioned samples was investigated. Compared with volumetric porosity, the porosity obtained from image analysis was slightly higher. Therefore, these results can represent the total porosity of pervious concretes. In addition, there is good consistency between porosity results obtained from CT images and from sectioned samples. These findings indicate that the porosity distribution among the samples produced for this study is relatively homogeneous.
- A comprehensive analysis can be performed using different fiber doses and types in future studies.
